# Isolation of Valuable Biological Substances from Microalgae Culture

**DOI:** 10.3390/foods11111654

**Published:** 2022-06-04

**Authors:** Olga Babich, Vyacheslav Dolganyuk, Anna Andreeva, Dmitriy Katserov, Liudmila Matskova, Elena Ulrikh, Svetlana Ivanova, Philippe Michaud, Stanislav Sukhikh

**Affiliations:** 1 Institute of Living Systems, Immanuel Kant Baltic Federal University, A. Nevskogo Street 14, Kaliningrad 236016, Russia; olich.43@mail.ru (O.B.); dolganuk_vf@mail.ru (V.D.); annaandreeva@kantiana.ru (A.A.); dkatze39@gmail.com (D.K.); liudmila.matskova@ki.se (L.M.); stas-asp@mail.ru (S.S.); 2 Department of Bionanotechnology, Kemerovo State University, Krasnaya Street 6, Kemerovo 650043, Russia; 3 Institute of Agroengineering and Food System, Kaliningrad State Technical University, Soviet Avenue 1, Kaliningrad 236022, Russia; elen.ulrich@mail.ru; 4 Natural Nutraceutical Biotesting Laboratory, Kemerovo State University, Krasnaya Street 6, Kemerovo 650043, Russia; 5 Department of General Mathematics and Informatics, Kemerovo State University, Krasnaya Street 6, Kemerovo 650043, Russia; 6 Institut Pascal, Université Clermont Auvergne, CNRS, Clermont Auvergne INP, F-63000 Clermont-Ferrand, France

**Keywords:** microalgae, proteins, carbohydrates, lipids, *Chlorella*, microalgae, fatty acid

## Abstract

Methods for purifying, detecting, and characterizing protein concentrate, carbohydrates, lipids, and neutral fats from the microalgae were developed as a result of research. Microalgae were collected from natural sources (water, sand, soil of the Kaliningrad region, Russia). Microalgae were identified based on morphology and polymerase chain reaction as *Chlorella vulgaris* Beijer, *Arthrospira platensis* Gomont, *Arthrospira platensis* (Nordst.) Geitl., and *Dunaliella salina* Teod. The protein content in all microalgae samples was determined using a spectrophotometer. The extracts were dried by spray freeze drying. Pressure acid hydrolysis with 1% sulfuric acid was determined to be the most effective method for extracting carbohydrates from microalgae biomass samples. The highest yield of carbohydrates (more than 56%) was obtained from *A. platensis* samples. The addition of carbohydrates to the cultivation medium increased the accumulation of fatty acids in microalgae, especially in *Chlorella*. When carbohydrates were introduced to nutrient media, neutral lipids increased by 10.9%, triacylglycerides by 10.9%, fatty acids by 13.9%, polar lipids by 3.1%, unsaponifiable substances by 13.1%, chlorophyllides by 12.1%, other impurities by 8.9% on average for all microalgae. It was demonstrated that on average the content of myristic acid increased by 10.8%, palmitic acid by 10.4%, oleic acid by 10.0%, stearic acid by 10.1%, and linoleic acid by 5.7% in all microalgae samples with the addition of carbohydrates to nutrient media. It was established that microalgae samples contained valuable components (proteins, carbohydrates, lipids, fatty acids, minerals). Thereby the study of the composition of lipids and fatty acids in microalgae, as well as the influence of carbohydrates in the nutrient medium on lipid accumulation, is a promising direction for scientific research in the fields of physiology, biochemistry, biophysics, genetics, space biology and feed additive production.

## 1. Introduction

Humans consume microalgae for centuries [1]. Microalgae have been exploited economically for the production of food products that are beneficial to human health since the 1950s and 1960s, particularly in the Far East [2]. Initially, interest in the cultivation of microalgae was associated with the plant-like characteristics of many microalgae, i.e., their ability to use CO_2_ as their sole source of carbon and sunlight as their sole source of energy. Microalgae are known to be substantially more efficient in photosynthesis than higher plants. They can reach 3–5% photosynthetic activity, which is higher than photosynthetic efficiency of agricultural plants [3]. Japanese commercial microalgae industry has developed around green algae of the genus *Chlorella* [4]. This paved the way for the development of a microalgae industry, which has thrived in Japan and other Far Eastern countries to this day. In the 1970s and 1980s, the major products of biotechnology were pigments (phycocyanin and carotenoids) and microalgae as raw materials for aquaculture. Active research has identified a wide range of bioactive compounds in microalgae. It demonstrated that microalgae can be used to produce protein, polyunsaturated fatty acids, vitamins, minerals, and carbohydrates [5,6]. Through antioxidant properties, anti-inflammatory, antimicrobial, and antiviral effects, these compounds may play an important role in cattle, poultry, shellfish, and fish feed, as well as human functional foods, contributing to the prevention of stomach ulcers, constipation, anemia, diabetes, and hypertension [7,8].

The rising demand for meat from an expanding population will be especially intense in the coming decades. Therefore, the traditional animal feed soybean needs to be substituted because otherwise it will occupy more and more arable land. Protein complexes derived from microalgae biomass may be used to make functional foods (dairy, desserts, pasta) in the future [9]. In comparison to proteins from other crops, algae proteins and their extraction are a comparatively unexplored area. *Spirulina* (*Arthrospira platensis*) biomass, for example, is a good source of polyunsaturated fatty γ-linolenic acid, which is vital for healthy human nutrition [10]. Recently, there has been increased interest in microalgae fats due to the fact that microalgae are being studied as a source of biofuels [11]. Extensive research is being conducted to enhance the content of neutral fats and triacylglycerols (TAGs). They are a source of methyl esters, which are produced from TAG during the transesterification process in the presence of catalysts and can be used in diesel engines [12].

Due to their oligosaccharide content, microalgae have a prebiotic role in maintaining a healthy microbiota composition in the human/animal gastrointestinal system. Normal human or animal gut microbiota cannot ferment microalgae oligosaccharides (at least not completely). However, these oligosaccharides selectively stimulate the growth and activity of certain beneficial bacteria (for example, Lactobacilli and Bifidobacteria), thus acting as prebiotics [13].

The list of beneficial properties of functional foods containing microalgae is constantly expanding [14,15]. The apparent inconsistency of some of the results may be due to differences in geographic origin, harvest period, (aquatic) environment, genetic variability, post-harvest conditions and extraction methods (including the type of solvent used) [16].

Typically, purification is the first step in the processing of accumulated microalgae biomass. Several methods of purifying biomass and microalgae extracts are currently under consideration (separation or centrifugation; filtration; flocculation; flotation). During separation and centrifugation, the cell suspension is exposed to centrifugal force and settles on the centrifuge walls. During separation, the liquid moves between rotating conical plates. In the process of rotation, biomass particles are thrown away from the center of rotation, slide along the conical plates to the periphery, forming a layer of condensed biomass near the inner wall of the vessel, while the clarified liquid moves upwards. The precipitated cells remain alive and retain their biochemical properties [7,17,18]. Centrifugation and separation are high energy consuming processes. To separate a substantial quantity of suspension under industrial microalgae cultivation conditions, a considerable number of pieces of equipment may be required. Filtration is the retention of suspended particles by a porous membrane under the action of a liquid pressure difference before and after the membrane [7,17]. Filtration is called sieving when particles are trapped by the membrane itself, and the size of the pores in the membrane is less than the size of the particles. If the size of the holes in the membrane is larger than the particle size, such filtration is called through the sediment layer—the first particles slip through the membrane, and then a layer of sediment is formed on the membrane, which serves as a filter. Filtration is a time-consuming and energy-intensive operation that necessitates the replacement of filter baffles on a regular basis. Flocculation is a process based on the physiological ability of microorganisms to settle during cultivation. It cannot be identified with cell sedimentation, which is a physical process [19]. The organization of the flocculation process involves the use of precipitating agents. The disadvantage of this method is that the coagulants contain toxic metal ions and also change the pH of the culture liquid, making it unsuitable for reuse. Difficulties can arise when using flocculants, since, in addition to microalgae cells, they can flocculate suspended particles and components of the nutrient medium present in the culture liquid. The presence of ballast substances in the resulting thickened suspension will complicate its further processing. Microorganisms, such as bacteria, diatoms, and others can act as bioflocculants. Their use is limited, since bioflocculating organisms require the introduction of additional nutrients into the culture liquid, and also can infect the microalgae monoculture itself. The process of autoflocculation (spontaneous sedimentation of microalgae) can occur when environmental conditions change, for example, an increase in the pH of the medium, a change in osmotic pressure, etc. In addition, the efficiency of flocculation can be increased when autoflocculating microalgae are introduced into the suspension, which do not require the introduction of additional chemical reagents and special cultivation conditions. It should be mentioned that the process of flocculation is still poorly understood. Flotation is the separation of microbial cells from culture liquid by their adhesion (sticking) to rising gas bubbles, the collection of foam, and its condensation. The collected foam settles and is drained as a result [7]. According to the method of organization, bubbling flotation, pressure flotation, and electroflotation are distinguished. When organizing flotation, frequent replacement of worn electrodes is required. In addition, electroflotation is an energy-intensive process.

Extraction methods are used in microalgae biotechnology to extract lipids, polypeptides, pigments, and other intracellular components [5,7]. Water, alkali and acid aqueous solutions, and organic solvents are used as extractants, depending on the type of extracted substance. Despite the existing methods of processing and purification of microalgae, the composition and properties of microalgae are still mostly unknown. One of the key initiatives to promote microalgae to the food market is the identification of components of the chemical composition of microalgae, as well as the optimization of methods for extracting valuable substances from them. Their lysis is one of the main drawbacks limiting the exploitation of numerous intracellular metabolites. It is then necessary to develop approaches to break microalgae cell walls. Their thickness and composition depend on the species of microalgae, growth conditions, and growth stage [17].

This research aimed to develop methods for purification, detection, and characterization of protein concentrate, carbohydrates, lipids, and neutral fats from a variety of *Chlorella vulgaris*, *Arthrospira platensis* and *Dunaliella salina*. The parameters of microalgae culture were studied, and the content of neutral fats was measured.

## 2. Materials and Methods

### 2.1. Reagents

All chemicals (NaOH, HCl, trichloroacetic acid, phosphate salts, and ultrapure water) used in the study were from Sigma Aldrich (Sigma-Aldrich Rus, Moscow, Russia). All chemicals were ACS grade reagents. All solutions were prepared using purified, deionized MilliQ water (MilliporeSigma, Burlington, WY, USA).

### 2.2. Microalgae Identification

Microalgae were collected from natural sources (water, sand, soil) in the period from October 2020 to December 2020 in various regions of the Kaliningrad Oblast Lake Vištytis (54°25′37″ N 22°43′30″ E), Lake Chaika (56°03′49″ N 29°04′50″ E), Lake Yantarnoye (56°01′44″ N 30°44′03″ E), Curonian Lagoon (55°07′00″ N 21°01′00″ E), Strait of Baltiysk (59°43′ N 28°24′ E), Baltic Sea coast (54°42.4′0” N 20°30.4′0” E), Lake Krasnoye (54°25′59″ N 22°30′27″ E)). They were sampled with a box-shaped bottom sampler, developed by the Institute for Biology of Inland Waters of the Russian Academy of Sciences (IBIW) (Borok, Russia), covering a square area of the bottom 160 × 160 mm in size with a maximum immersion depth of 440 mm in bottom sediments. Immediately after transportation to the shore, samples were collected using plastic tubes with an inner diameter of 45 mm. The tubes were sealed at both ends and stored in an upright position at +4 °C. In the laboratory, the core was cut lengthwise and halved using two thin stainless steel plates inserted into the cut. The core halves were then divided into transverse samples (slices) with a step of 5–10 mm [18]. All samples were stored at −20 °C in the dark, in plastic bags with squeezed air from which samples of microalgae were taken for research.

After being isolated from natural sources and transferred to culture media, cultured and purified microalgae were identified using morphology [20,21] and the polymerase chain reaction (PCR).

Genetic identification of the obtained cultures of microalgae and cyanobacteria was carried out by sequencing 16s and 18s rRNA gene fragments using the Sanger method. PCR mixtures were prepared in a volume of 50 μL and included: 0.2 uM forward and reverse primers, master mix qPCRmix-HS SYBR (Evrogen, Moscow, Russia,) and ~10–30 ng of DNA for one reaction (Table 1). Fragment amplification was carried out in a CFX-96 thermal cycler (BioRad, Berkeley, CA, USA) according to the following program: 95 °C—3 min ≥ 28 cycles (95 °C—30 s; 55–60 °C—30 s; 72 °C—30 s) ≥ 72 °C—3 min. The PCR products were sized and purified by preparative gel electrophoresis using the Cleanup Mini kit (Evrogen, Moscow, Russia). Two-way sequencing in the directions (5′-3′) and (3′-5′) was performed on a 3500 genetic analyzer (Applied Biosystems, Wakefield, MA, USA).

Two-way sequencing in the directions (5′-3′) and (3′-5′) was performed on a 3500 genetic analyzer (Applied Biosystems, Wakefield, MA, USA). Further sample preparation for sequencing incorporated BrilliantDye™ Terminator (v 3.1) (NimaGen BV, Nijmegen, The Netherlands) chemistry with the following cycle sequencing reaction conditions: 96 °C—2 min ≥ 35 cycles (96 °C—10 s; 52 °C—15 s, 60 °C—3 min) ≥ 72 °C—1 min ≥ 95 °C—15 s. Forward and reverse primers from template generation were used in separate cycle sequencing reactions to achieve two-way sequencing. Products were purified and sequenced on 3500 Genetic Analyzer (Applied Biosystems, Wakefield, MA, USA). Acquired sequences with reviewed, edited and assembled using “BioEdit 7.1”. Results were used as query sequences for BLAST with default settings.

### 2.3. Microalgae Cultivation

The stock cultures were grown in 250 mL conical flasks (working volume 100 mL) under white-red light (distance from the lamp 30 cm) at room temperature (24 °C), at a light power of 20 W, a light intensity of 50–60 µmol photons m^−2^ s^−1^, and a light/dark cycle of 12 h/12 h. Once the stock cultures had reached the exponential growth phase, they were used to inoculate 100 mL of appropriate nutrient medium supplemented with up to 3.0% carbohydrates (maltose) to boost the growth and reproduction of microalgae with a high carbohydrate content. Optical density (OD) at a wavelength of 750 nm was 1.0, measured on a PE-5400UF spectrophotometer (Vikon-service, Astrakhan, Russia). *Chlorella* was cultured using Bold Basal Medium [19,25], *Arthrospira*—Zarrouk’s medium [8] and *Dunaliella*—Ramaraj Medium [26].

*Arthrospira platensis*, *Dunaliella salina* and *Chlorella vulgaris* were grown with constant stirring on an orbital shaker Unimax 1010 (Heidolph Instruments GmbH & Co. KG, Schwabach, Germany) at 65 rpm, at T = 22 °C, for seven days, until a density of 10^6^–10^8^ cells/mL.

### 2.4. Measurement of Soluble Protein Concentration

The soluble protein content of all samples was determined using a SolidSpec-3700/3700 DUV spectrophotometer (Shimadzu, Kyoto, Japan) by the Bradford method using a calibration curve based on BSA in 0.5 M NaOH solution (Kompaniya Pushchinskiye laboratorii, Moscow, Russia) [17], according to the manufacturer’s instructions, at a wavelength of 595 nm. The soluble protein complex (the total of soluble proteins of all fractions having different molecular weights) was determined in 25 mL of the extract. The protein content in the measured fractions was determined in relation to the dry mass, as a percentage:

$$\mathrm{Solubilized}\;\mathrm{material},\;\%=\frac{w_{0}-w_{1}}{w_{0}}\times 100$$

where *w*_0_—initial sample quantity (2 g), and *w*_1_—weight of the dried precipitate after extraction.

The dissolved protein content was compared to the crude protein content in the sample on absolute dry matter (a.d.m.) using the Kjeldahl method. The method is based on the mineralization of organic matter with sulfuric acid in the presence of a catalyst, which results in the formation of ammonium sulfate, the destruction of ammonium sulfate with alkali, which results in the release of ammonia, and the stripping of ammonia with water vapor into a sulfuric or boric acid solution, followed by titration. Then, the mass fraction of nitrogen and crude protein content were calculated (by multiplying by a factor of 6.25) [22].

### 2.5. Extraction of the Protein Complex

**Figure 1:**
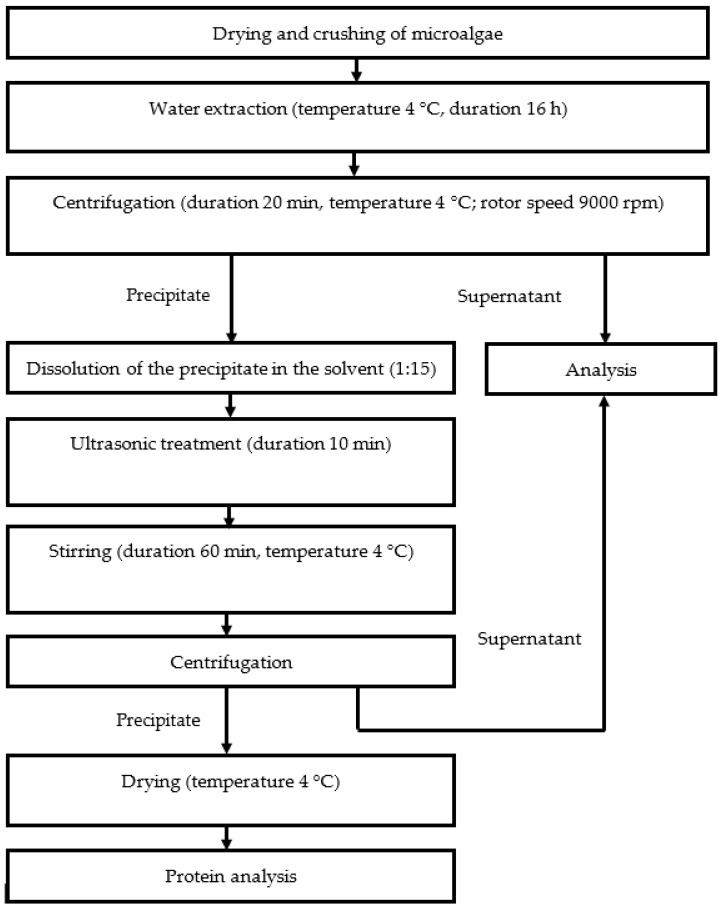
Protein extraction flowchart.

A standard protein extraction flowchart is presented in Figure 1. Protein extraction from microalgae was performed using the modified method of Harnedy and FitzGerald [22].

The microalgae biomass was kept at T = 40 °C in an oven (Memmert, Schwabach, Germany) for 18 h. Then, the samples were stored at T = 4 °C. Two g of dried algae were suspended in 40 mL of distilled water at T = 4 °C for 16 h. Microalgae were precipitated by centrifugation at 9000 rpm for 20 min at T = 4 °C (Thermo Scientific™ Heraeus™ Megafuge™ 16 Centrifuge Series, Waltham, MA, USA). Hydrochloric acid and caustic alkali were added in increasing concentrations from 0.1 to 0.4 M to the resulting precipitates, stirred, and a precipitate was obtained. The precipitate was dried to a constant weight and stored in an airtight container.

Precipitation was observed again after a portion of the precipitate was dissolved in 0.1 M HCl solution. The precipitate was then dissolved in 0.1 M NaOH solution, stirred, and precipitated for the third time. Supernatants obtained after acid and alkaline extraction were combined. The precipitate was dried to a constant weight and stored in an airtight container.

After the second acid-base extraction, a portion of the dried precipitate was redissolved. Proteins were extracted from dissolved microalgae precipitates by switching the order of solvent additions: 0.1 M NaOH solution first, then 0.1 M HCl solution. Precipitation was observed. The precipitate was dried to a constant weight and stored in an airtight container.

A portion of the microalgae precipitate after the third acid–base extraction was dissolved either in a 0.1 M HCl solution or in a 0.1 M NaOH solution, and the resulting mixtures were sonicated for 10 min. Soniprep 150 plus (Soniprep Limited, Heathfield, UK) with a power of 750 W and a frequency of 20 kHz was used; amplitude levels from 22.8 to 68.4 µm. Probe diameter 13 mm.

A weight to volume ratio of 1:15 was employed for all extraction conditions, and the same conditions were employed for precipitate resuspension for 1 h at T = 4 °C and precipitate sedimentation by centrifugation at 9000 rpm for 20 min at T = 4 °C. The precipitates were dried at T = 40 °C for 72 h.

### 2.6. High Performance Size Exclusion Chromatography (HPSEC)

Proteins in the resulting extracts were analyzed using a Waters^®^ Alliance HPLC High Throughput (HT) system (Waters, Milford, MA, USA) equipped with a sample manager, column heater (T = 100 °C), and a photodiode array detector at an absorption wavelength of 214 nm. A ZORBAX macroporous HPLC column (GF-250/450, Agilent Technologies, USA) with a particle size of 4–6 µm and a pore size of 150–300 Å was used. A phosphate buffer with pH 7.5 was used as the mobile phase. ten μL of sample were injected into the system. Based on the literature [18], a flow rate of 0.85 mL/min was maintained in the column at 40 °C for 25 min. A calibration curve was constructed using albumin (66 kDa), carbonic anhydrase (29 kDa), cytochrome C (12.4 kDa), aprotinin (6.5 kDa), angiotensin II acetate (Asp-Arg-Val-Tyr-Ile-His-Pro.-Phe; 1046 Da), and leucine-enkephalin (Tyr-Gly-Gly-Phe-Leu; 555 Da).

The average molecular weight of proteins was determined using Capel-105 capillary electrophoresis (Lumex, St. Petersburg, Russia) [5].

### 2.7. Enzymatic Hydrolysis of Microalgae

During enzymatic hydrolysis, the cell wall of microalgae was destroyed by the action of different types of enzyme preparations: cellulase (Cellucast^®^), xylanase (Shearzyme^®^), β-glucanase (Ultraflo^®^), κ-carrageenase, β-agarase. Enzymes κ-carrageenase and β-agarase were purchased from DV-Ekspert, Moscow, Russia. Enzymatic hydrolysis was carried out in mild conditions for 1 h at atmospheric pressure and under the following additional conditions: for amylase, the optimum temperature was 50 °C, the operating temperature 30–70 °C, the optimum pH 4.7, the operating pH 3.0–7.0; for protease and α-gluconase, the optimum temperature was 50 °C, the operating temperature 45–60 °C, the optimum pH 5.7, the operating pH 5.7–6.0; for cellulase, β-glucanase, xylanase, glucoamylase, the optimum temperature was 60 °C, the operating temperature 50–70 °C, the optimum pH 4.7, the operating pH 4.5–7.0. The ratio of raw material:extractant was 1:45. 1000 g of microalgae biomass, 45 L of extractant were used, cellulase concentration was 0.5%, xylanase concentration was 0.5%, β-glucanase concentration was 0.8%, κ-carrageenase and β-agarase concentration was 0.6%. Alcohol, water, glycerin, propylene glycol, etc. were used as extractants. The listed reagents were used in combination for a more complete hydrolysis process. After which the hydrolysis product was dried in an Inei-6 lyophilic dryer (Vilitek, Moscow, Russia) until the volume was reduced by a factor of 2, and extraction was performed.

### 2.8. Potter Homogenization of Microalgae

Potter homogenization of microalgae was carried out using a Potter homogenizer, which is a thick-walled test tube with a ground glass pestle, rotated by a Tecnal model TE-099 electric motor (TEE, Piracicaba, Brazil). The internal dimensions of the Potter homogenizer were 30 × 35 × 54 cm. Dry algae were added in an amount of 0.5 g for every 36 mL of solvents at room temperature. This mixture was processed in the Potter homogenizer at medium speed for 3 min at room temperature. The sample was then centrifuged at 3500 rpm for 8 min at 4 °C. The acid and alkali were carefully collected and discarded and the microalgae were used for subsequent studies.

### 2.9. Osmotic Stress of Microalgae

The variability of microalgae cell volumes under osmotic stress was studied. Algae from the stock culture were inoculated on media enriched with biogenic components at the exponential stage of growth 35.0. (I35.0, hyperosmotic conditions). The inoculum titer was 2 × 10^4^ cells/mL. The algae were grown for 35 days. The number of cells and cell sizes in the samples were determined immediately after inoculation and then every 2–3 days after that. The number of cells was estimated by direct counting in the Nageotte chamber. The cell volumes were calculated by the method of geometric similarity [18] based on measurements of the linear dimensions of at least 50 cells. Cell sizes were determined after fixation with Lugol’s solution from their image using the camera software DCM 300 (Hangzhou Huaxin IC Technology Inc., Zhejiang, China) mounted on a fluorescent microscope.

### 2.10. Deep Freezing of Microalgae

Deep freezing of microalgae was carried out in a device for cultivating microalgae, which contained a container, a light source, and a gas supply system.

The algae were subjected to deep freezing at a temperature from −20 to 196 °C for 10–30 min, then they were thawed and placed in a nutrient medium for one week. Next, the living tissue of the algae was crushed, the resulting cell aggregates were placed in a nutrient medium and cultivated for 3 weeks with constant stirring, aeration, and weekly renewal of the nutrient medium. Liquefied carbon dioxide with a freezing point of −56.6 °C was used as cryogas. Deep freezing leads to irreversible denaturation of dissolved proteins, damage to cytoplasmic membranes, as well as damage to intracellular contents due to the development of intracellular crystallization. Dehydration of cells due to freezing leads to an increase in the concentration of osmotically active substances in the cell.

### 2.11. Purification of Protein Complexes from the Solvent by Freeze Drying

The protein complex in a volume of 100 mL was placed in a 200 mL round bottom flask. The purification process was carried out in a vacuum of 127 mbar, at a temperature of 40 °C for 30 min. The volume of the extract was reduced by 30%. Extract samples were analyzed by HPLC.

The protein complex was poured into 10 mL Falcon tubes and freeze dried for 24 h. The volume of liquid extracts decreased by 77%. The lyophilized complex was in the form of a dark colored solution. Extract samples were analyzed by HPLC.

It was determined that a smooth temperature transition from the temperature of the protein eutectic (T = 31 °C) to room temperature 22 ± 0.2 °C, followed by a temperature transition to 0 °C is required to preserve the initial composition of the extracted protein complex. Furthermore, the time of cooling to 0 °C should not be shorter than 12 h. In order to avoid losses in the composition of the protein complex, we reduced the intensity of freeze drying. In the first step of drying, the vacuum in the chamber was lowered to 30–35 Pa until the temperature reached the eutectic threshold. The vacuum was gradually increased after that.

### 2.12. Measurement of Carbohydrates

The concentration of total carbohydrates was determined by the phenol-sulfuric acid method [19]. A 5% (*w*/*v*) solution of phenol was prepared in distilled water. Fifty µL of test samples and 2 mL of concentrated sulfuric acid were added to 50 µL of a phenol solution. The solution was left at room temperature until it turned orange. A_490_ was measured. The d-glucose concentration scale was used to construct the standard curve.

### 2.13. Mixotrophic Cultures of Microalgae

The effect of introducing carbohydrates (glucose, maltose, and fructose) on the microalgae culture growth rate and fat accumulation under mixotrophy was studied. Carbohydrates were added to culture media to a final concentration of 0.1 g/L. For each microalgae/carbohydrate pair, inoculation was performed with repetition in three flasks (total 27 flasks). One mL samples were obtained during culture growth and then preserved in a formaldehyde solution at a final concentration of 1.1%. The samples were stored at +4 °C. The measurements were carried out for 30 days. A_600_ was used to determine the microalgae culture mass production. To assess the fat content, aliquots of microalgae cultures were stained with the fluorescent dye Bodipy, which specifically binds to neutral fats. The fluorescent signal was recorded at 525 nm. The level of chlorophyll in microalgae cells was estimated by A_750_ measurements. Measurements were taken in a Clariostar plate spectrophotometer with three repetitions, in aliquot volumes of 150 μL. Each canned sample was actively combined on a vortex before being resuspended with a pipette 20–40 times until a homogenous solution was formed. Dispenser resuspension was critical for homogenization of *Spirulina* culture samples. Next, aliquots of 150 µL were transferred into the wells of the plate.

### 2.14. Isolation of Lipids

Extraction of total lipids was performed based on the Folch method [25]. For this, 2 mL of a mixture of chloroform:methanol (2:1 by volume) per 100 mg of dry biomass was used. Next, the sample was treated with ultrasound for 30 min. Then 0.25 volumes of 0.9% NaCl solution were added to the sample and the mixture was intensively stirred. After phase separation, the organic/lower phase was separated and evaporated using a rotary evaporator to constant weight. The dry weight of the lipid fraction was determined. The lipid content *W_L_* (%) was determined using the following formula:

$$W_{L}=\frac{m_{L}}{m_{b}}\cdot 100\%$$

where *m_L_*—mass of extracted lipids; *m_b_*—mass of dry biomass.

### 2.15. Determination of Fatty Acid Composition

The composition of lipids was determined using high performance GC/MS instrument Agilent 5977B (Agilent Technologies, Inc., Santa Clara, CA, USA) using a mass spectrum of 50–800 m/z. The ionization source used was an electron impact, the sensitivity of the mass spectrometer was 0–12 g in different scanning modes, the GC/MS instrument supported a selective search for the specified groups of particles (SIM mode), and also performed a full scan in the specified range (Full scan mode). Column and elution chromatographies were employed; a mixture of n-hexane/ethyl acetate was used as a mobile phase (eluent), polyethylene glycol, esterified with nitroterephthalic acid, was used as fixed phase. Column parameters: polarity 58, temperature range from 40 to 250 °C, column length 15 m, column inner diameter 0.25 mm, stationary phase layer thickness 0.25 µm. The heating of the thermostat was carried out in a gradient mode according to the following program [13]: 0 min—80 °C; 10 min—150 °C; 30 min—250 °C. Injection volume was 1 µL.

Transesterification of fatty acid triglycerides was carried out with freshly prepared sodium methoxide solution. 1.15 g of metallic sodium was dissolved in 25 mL of methanol, the solution was cooled to room temperature before use. In a 2 mL polypropylene tube, 1.9 mL of hexane was introduced, followed by 10 μL of the studied lipid fractions. Then 100 μL of freshly prepared sodium methoxide solution was added. The mixture was intensively stirred for 1 min on a vortex, kept for 10 min at room temperature, and filtered through a 0.40 µm syringe filter into a vial for subsequent chromatography.

### 2.16. Staining of Neutral Lipids

Neutral fats in microalgae cells were stained with the fluorescent dye Bodipy, which specifically binds to neutral fats [13]. Microalgae samples were collected at regular intervals once a day under sterile conditions, formalin was added to a final concentration of 1.1%, and the samples were kept at 4 °C.

Staining microalgae was carried out for 1 h at 25 °C on a rotary shaker. Microalgae were collected by centrifugation at 4000× *g*, 15 min. The pellets were washed twice in 1xPBS buffer. The fluorescent signal was recorded in a Clariostar plate spectrophotometer with 3 replicates in aliquot volumes of 150 μL, with the values Excitation = 477 − 14 nm and Emission = 525 − 30 nm.

Preparation of microalgae for confocal microscopy included additional staining of nuclei with Hoechst 33,342 stain (Thermo Fisher Scientific, Waltham, MA, USA): 100 µL of microalgae cultures were made up to 500 µL with 1x PBS. One μL of Hoechst 33,342 was added to the samples to a final concentration of 5 μg/mL and Bodipy (Pushchin Laboratories Company LLC, Moscow, Russia) to a final concentration of 2.5 μg/mL; incubated for an hour at 25 °C on a rotary shaker; washed twice in 500 μL of 1x PBSbuffer by centrifugation at 4000× *g* for 30 min. Finally, 100 µL of glycerol were added added to pellets. To fix the cells on the slide before microscopic examination, 10–30 µL of the cell suspension was mixed with 100 µL of histological medium (Mowiol 4-88, Tris-Cl, glycerol). Images were obtained using an NS-3500 laser confocal scanning microscope (Promenergolab, Moscow, Russia).

Stained preparations were stored in an aluminum envelope at +4 °C. The shooting was carried out using ×63 and ×100 lenses. The growth of microalgae cultures in media without the addition of carbohydrates was used as a control for assessing lipid content.

### 2.17. Polysucrose Decomposition

Polysucrose was decomposed by enzymatic hydrolysis using the Allzyme BG enzyme (Pushchin Laboratories Company LLC, Moscow, Russia) at a temperature of 40 °C, pH 4.7, duration 24 h.

### 2.18. Crude Ash Determination

A 1–2 g microalgae sample was used to determine the mineral components on an analytical balance in a calibrated crucible. The crucibles with the sample were placed in a UED-16-12D muffle furnace (Uedgroup, Moscow, Russia) and the sample was burned for 30 min at a low-red heat temperature (500–525 °C). After the crucibles ceased to smoke, they were taken out and cooled in air. Then 6–8 drops of concentrated nitric acid were added to each crucible. The crucibles were again placed in a muffle furnace heated to 800–1000 °C. After combustion, the crucibles were placed in a desiccator and weighed on an analytical balance after 40–60 min. The content of crude ash in the test substance in percent was calculated using the formula:

$$X=\frac{a\cdot x\cdot 100}{n}$$

where *x*—percentage of crude ash in plant material; *a*—ash weight, g; *n*—weight of absolute dry matter, g.

### 2.19. Statistical Analysis

Standard mathematical statistics methods were used to process the data. Each experiment was performed in three replicates. The gathered data were expressed as means ± standard deviation. The correspondence of the used samples to the normal distribution was assessed using the t-test (mathematical expectations) for independent samples and Fisher’s test (variance). The Levene test was used to ensure that the variances of the isolated samples were equal. The results were analyzed using Duncan multiple range test at *p* < 0.05 to identify samples that were significantly different from each other. Data were subjected to analysis of variance (ANOVA) using Statistica 10.0 software (StatSoft Inc., 2007, Tulsa, OK, USA).

## 3. Results

### 3.1. The Microalgae Identification

Analysis of the 18S rRNA gene sequences revealed that the following microalgae were isolated from natural sources: *Chlorella vulgaris* and *Dunaliella salina*; the 16S rRNA gene sequence revealed that the cyanobacteria *Arthrospira platensis* were isolated. Primary denaturation of primers was carried out. The resulting sequences were loaded into GenBank with accession numbers ON514161 and ON514162. Based on comparative external morphological features, the strains of these microalgae and cyanobacteria were determined as C-11 *Chlorella vulgaris* Beijer, C-38 *Chlorella vulgaris* Beijer, C-66 *Chlorella vulgaris* Beijer, B-256 *Arthrospira platensis* Gomont, B-287 *Arthrospira platensis* (Nordst.) Geitl., D-294 *Dunaliella salina* Teod using Collections of microalgae and cyanobacteria IPPAS IPP RAS (USI CMC IPPAS IPP RAS,). BLAST analysis of the acquired 18S and 16s rRNA gene sequences revealed 99.0-99.5% identity to *Chlorella vulgaris*, *Dunaliella salina* and *Arthrospira platensis*. The resulting sequences were loaded into GenBank with accession numbers ON514161, ON514162 and ON521863.

### 3.2. Composition of Microalgae Isolates

Samples of microalgae extracts were obtained by chemical or physical extraction and enzymatic hydrolysis. The content of proteins in the extracts was determined by the Kjeldahl method and spectrophotometrically. The obtained data are presented in Table 2. There was no significant variation in protein content when measured using the Kjeldahl method or spectrometrically.

The characteristics of proteins, carbohydrates, and lipids extracted by chemical extraction with acid or alkali are presented in Table 3.

Characterization of proteins, carbohydrates, and lipids obtained by joint extraction with acid and alkali is presented in Table 4.

Proteins, carbohydrates, lipids content in terms of dry weight was found to be in the 3–15% range.

The effect of ultrasound (US) on the extraction of proteins, carbohydrates, and lipids is presented in Table 5.

### 3.3. Carbohydrate Extraction

**Figure 2:**
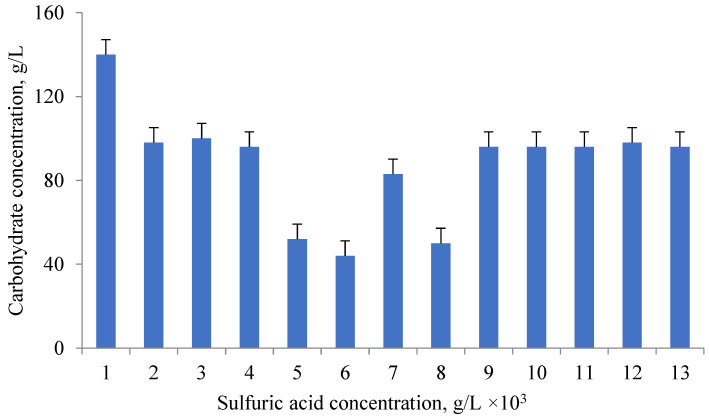
Carbohydrate (g/L) at various sulfuric acid concentrations during acid hydrolysis of 50 g/L of biomass.

The total amount of carbohydrates extracted from microalgae *D. salina* D-294 is presented in Figure 2.

### 3.4. The Influence of Carbohydrates in the Nutrient Medium on the Content of Neutral Lipids in Microalgae

**Figure 3:**
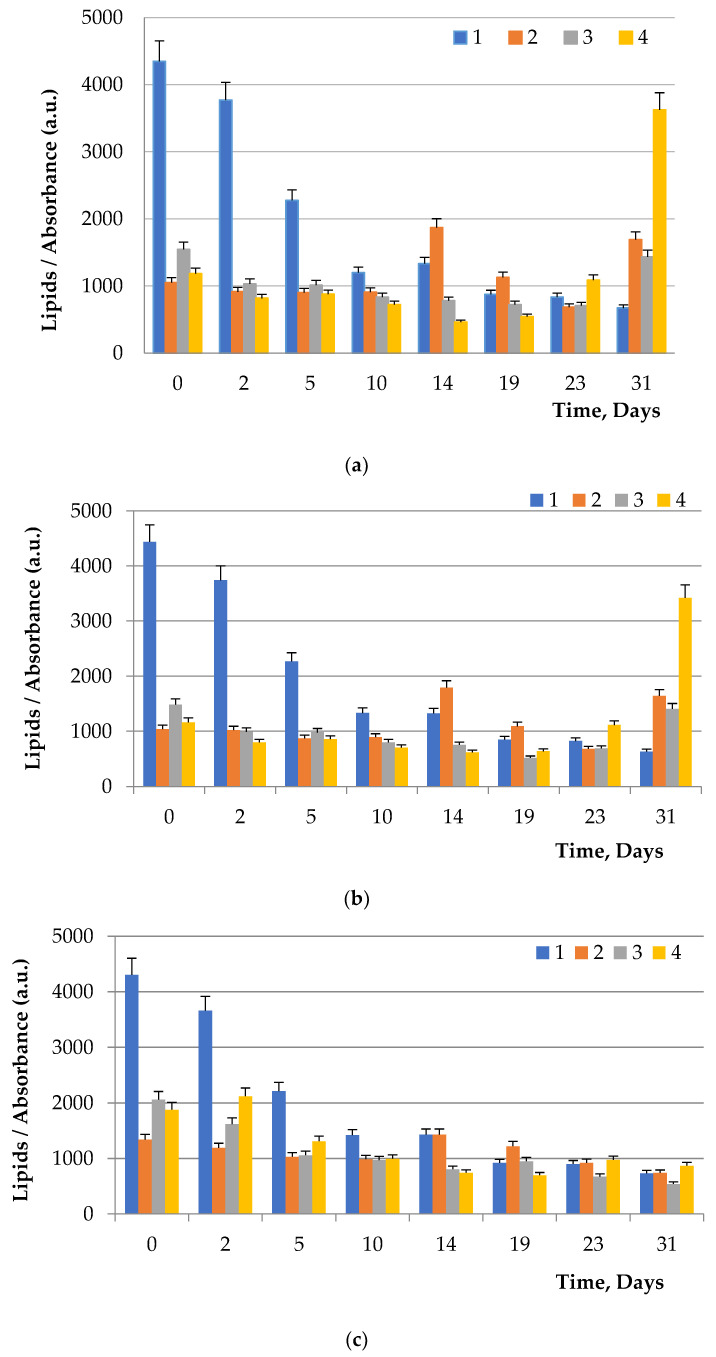

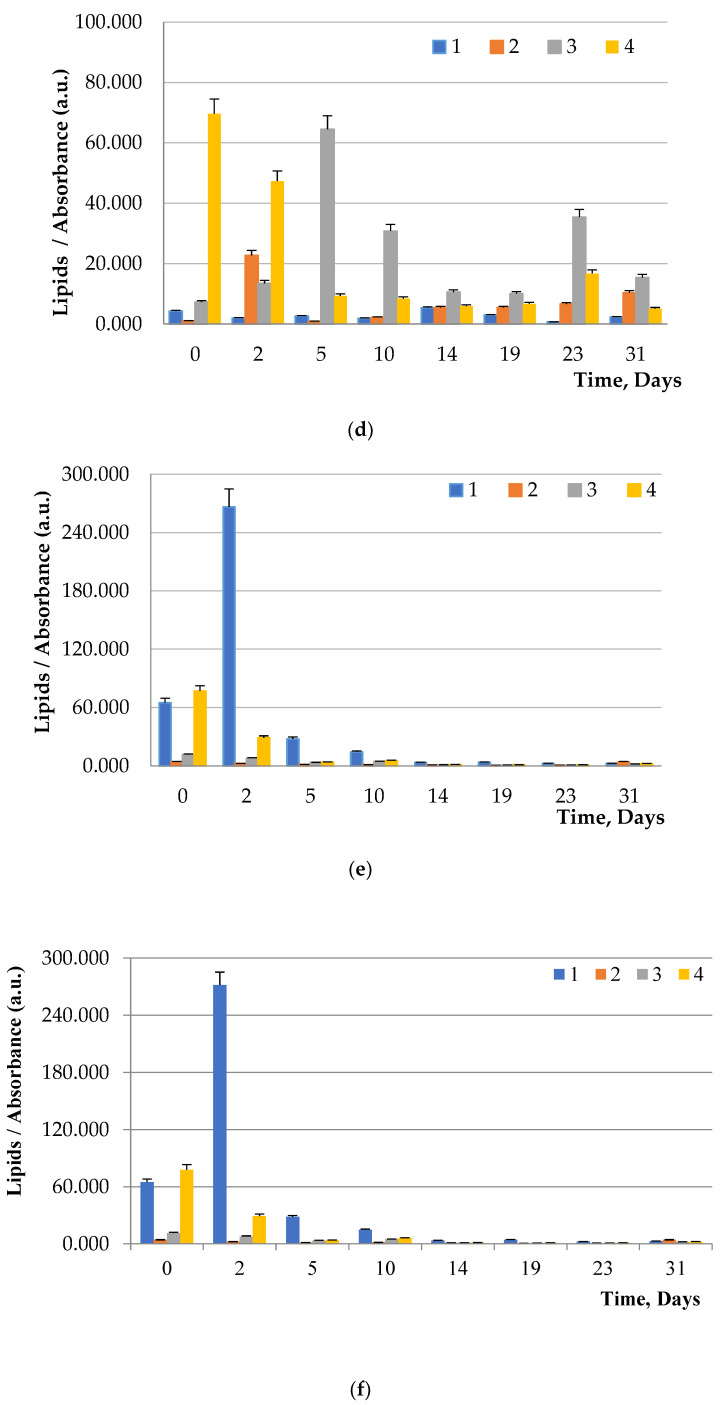
Influence of carbohydrates supplementation in culture media on lipid accumulation by (**a**) *Chlorella vulgaris* C-11, (**b**) *Chlorella vulgaris* C-38, (**c**) *Chlorella vulgaris* C-66, (**d**) *Dunaliella salina*, (**e**) *Arthrospira platensis* B-256 and (**f**) *Arthrospira platensis* B-287: 1—control; 2—glucose; 3—fructose; 4—maltose. The addition of carbohydrates was carried out under sterile conditions.

Figure 3 demonstrates the results of the effect of carbohydrates in the nutrient medium on the concentration of neutral lipids in microalgae. The effect of glucose, fructose, and maltose on the accumulation of lipids by microalgae *C. vulgaris* C-11, *C. vulgaris* C-38, *C. vulgaris* C-66, *D. salina*, *A. platensis* B-256, and *A. platensis* B-287 compared to control was studied. The control was the accumulation of lipids in microalgae cultivated without the addition of carbohydrates.

The chemical composition of microalgae samples after cultivation in a nutrient medium without and with the addition of carbohydrates (glucose, fructose, maltose) is presented in Table 6.

The lipid and fatty acids species composition of 100 g microalgae without and with the addition of carbohydrates is presented in Table 7 and Table 8.

**Figure 4:**
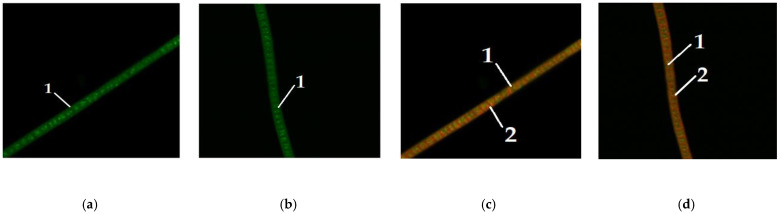
Microalgae *Arthrospira platensis* without the addition of carbohydrates to nutrient media (1—lipid droplet, 2—chlorophyll): (**a**,**b**) visualization of lipids; (**c**,**d**) visualization of chlorophyll.

Since the addition of carbohydrates had the greatest impact on the *Chlorella vulgaris* culture, a microscopic study of the content of neutral lipids in individual *Chlorella vulgaris* cells was performed. Figure 4 demonstrates lipid staining in microalgae *Arthrospira platensis* without the addition of carbohydrates to the nutrient media.

**Figure 5:**
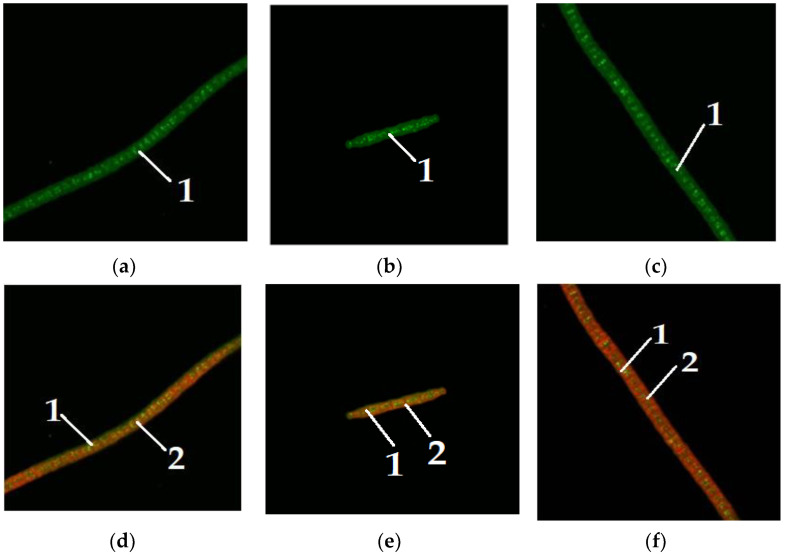
Microalgae *Arthrospira platensis* when glucose was added to the nutrient medium (1—lipid droplet, 2—chlorophyll): (**a**–**c**) visualization of lipids; (**d**–**f**) visualization of chlorophyll.

Figure 5 depicts the dye staining of lipids in microalgae *Arthrospira platensis* when glucose is added to the nutrient media.

**Figure 6:**
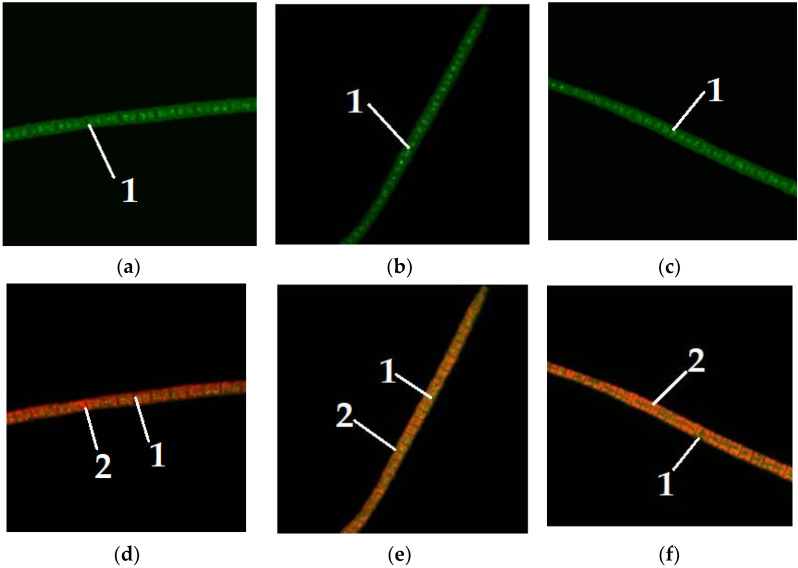
Microalgae *Arthrospira platensis* when maltose was added to the nutrient medium (1—lipid droplet, 2—chlorophyll): (**a**–**c**) visualization of lipids; (**d**–**f**) visualization of chlorophyll.

Figure 6 shows the staining of lipids with dyes in microalgae *Arthrospira platensis* when maltose is added to nutrient media.

**Figure 7:**
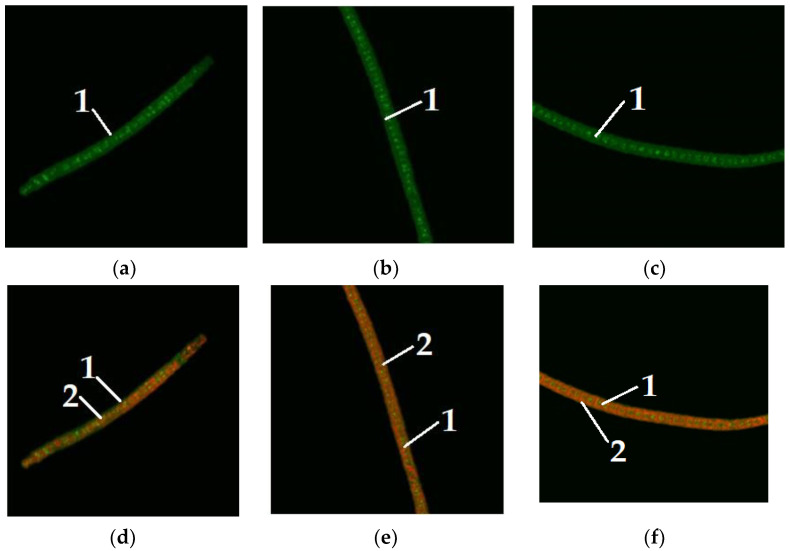
Microalgae *Arthrospira platensis* when fructose was added to the nutrient medium (1—lipid droplet, 2—chlorophyll): (**a**–**c**) visualization of lipids; (**d**–**f**) visualization of chlorophyll.

Figure 7 demonstrates the staining of lipids with dyes in microalgae *Arthrospira platensis* when fructose is added to nutrient media.

## 4. Discussion

*Arthrospira*, *Chlorella* and *Dunaliella* strains were isolated from the Baltic Sea, identified by morphology and using partial sequences of the 18S and/or 16S rRNA gene. Analysis of microalgae morphologies indicated that the culture of *C. vulgaris* was characterized by slightly ellipsoidal cells, 1.5 to 2.0 µm in size, green or dark green in color. The cells have no flagella. According to the analysis of morphological features obtained through microscopy of *A. platensis* this cyanobacteria has cells ranging from 8.0 to 10.0 µm in length and from 2.0 to 4.5 µm in width. Additionally, the cells were found to be slightly spiralized green or dark green trichomes. *D. salina* cells are broadly oval with a narrowing at one end. Cell size ranges from 7.5 × 10.5 µm. The cells are green and have flagella.

Acid, alkaline and acid-base hydrolysis of microalgae extracts were studied. The results of these types of hydrolysis were compared with additional exposure to ultrasound. It was found that the optimal conditions for the efficient extraction of carbohydrates from microalgae biomass was acid hydrolysis under pressure using 1% sulfuric acid. The highest yield of carbohydrates, more than 56%, was obtained from *A. platensis* samples. The influence of ultrasound on protein, carbohydrate, and lipid yields was not significant.

Figure 4 shows microalgae *A. platensis* of different colors, grown without the addition of carbohydrates to the nutrient medium. Figure 5, Figure 6 and Figure 7 show microalgae *A. platensis* grown with the addition of carbohydrates (glucose, fructose, maltose). The green background is nonspecific autofluorescence previously described by Tang and Dobbs [27]. Microscope channel settings: excitation—488 nm/emission—492–544 nm. Green inclusions are lipid drops after staining with BDP FL dye, an analogue of Bodipy FL (Lumiprobe GmbH). Microscope channel settings: excitation—488 nm/emission—492–544 nm. Red inclusions are chloroplasts. Microscope channel settings: excitation—633 nm/emission—647–722 nm. Cyanobacteria preparations were fixed in Mowiol 4-88 medium and examined under an LSM 780 laser scanning confocal microscope (Carl Zeiss Group, Oberkochen, Germany) with an EC “Plan-neofluar 100×/1.3 Oil” objective.

Only *A. platensis* was found to have high-quality and informative pictures that could be used to compare the buildup of lipid inclusions among the cyanobacteria and microalgae studied. In the case of *C. vulgaris* and *D. salina*, the resulting photographs did not provide a sufficiently clear picture of the location of fatty inclusions due to the small size of the cells and the insufficient capacity of the available equipment. The staining of microalgae *C. vulgaris* and *D. salina* showed a similar pattern, according to [28].

The protein recovery (%) increased gradually as the acid concentration in the extraction solutions increased. Concentrated alkaline solutions extracted the protein more efficiently than any concentration of hydrochloric acid (Table 3). Ultrasound (amplitude 68.4 µm) promoted more efficient protein extraction when alkaline solutions were used (Table 5). Sequential extraction using first acid and then alkali proved to be the most effective of all combinations studied. Ultrasound with amplitude levels of 22.8 µm and 68.4 µm was studied as an unconventional pretreatment method to increase protein. An ultrasound amplitude of 68.4 µm applied to the rehydrated microalgae sediment in an alkaline solution allowed obtaining a protein above 57%. The use of ultrasound allowed for the reduction of required acid or alkali concentrations as well as a significant reduction in extraction time (from 60 to 10 min). The use of ultrasound to improve traditional acid and alkaline extraction methods can be seen as an environmentally friendly alternative. The action of ultrasound can be explained by the processes of bubble cavitation, which contributes to the destruction of biological matrices, followed by the release of intracellular proteins. Thus, a greater protein can be obtained when using successive extraction with acid, then alkali, and then ultrasound. The difference between the initial weight of the microalgae samples and the weight of the sediment after extraction, in combination with the amount of protein recovered, suggests that non-protein compounds were also extracted (Table 6), which were determined by the phenol-sulphuric acid method (carbohydrates), or, based on the Folch method (lipids). The content of components (minerals, carbohydrates, proteins, lipids) was determined as described in Section 2.4, Section 2.5, Section 2.12 and Section 2.14.

Analysis of the data presented in Table 6 allows concluding that when carbohydrates were added to the nutrient medium, the content of carbohydrates in the samples of *C. vulgaris* C-11 extracts increased by 24%, *C. vulgaris* C-38 by 22.4%, *C. vulgaris* by 27.7%, *A. platensis* B-256 by 20.1%, *A. platensis* B-287 by 29.8%. However, in the samples of *D. salina* D-294, the content of carbohydrates decreased by 17.9%, which indicates that the increased content of carbohydrates in the nutrient medium inhibited the growth of microalgae biomass due to a decrease in the process of photosynthesis, which prevented the accumulation of carbohydrates in samples of microalgae extracts. When carbohydrates were added to the nutrient medium, the content of lipids in the extracts of microalgae *C. vulgaris* C-11 increased by 3.4%, *C. vulgaris* C-38 by 3.8%, *C. vulgaris* by 5.2%, *A. platensis* B-256 by 1.6%, *A. platensis* B-287 by 4.5%. The concentration of carbohydrates increased by 19.1% in *D. salina* D-294 samples, indicating that adding carbohydrates to the nutritional medium during microalgae growing aided in the buildup of lipids in microalgae extracts.

Carbohydrates are part of the microalgae cell walls. Cell wall disruption is a key factor in obtaining carbohydrate. Acid hydrolysis under pressure is one of the ways to destroy the cell walls of microalgae [29]. The effectiveness of different sulfuric acid concentrations and autoclave loading was examined. Carbohydrates of the cell walls of microalgae (such as plant cell walls) were isolated during acid hydrolysis under pressure at 121 °C for 20 min. Microalgae cells are similar in structure to plant cells, but they have specific features. The sulfuric acid content was varied from 0.1 to 5% (*v*/*v*), as well as the autoclave loading (10–50 g/L). The reaction temperature and time for 10 g microalgae biomass were chosen according to the literature data [29]. The of glucose in glucose equivalent was close to 100% when using a sulfuric acid concentration of 2% or higher; at a concentration of 1%, the yield was 96%. 1.67 g/L of carbohydrates were produced at a 2% acid concentration and a minimum autoclave load of 10 g/L. A glucose of more than 95% is achieved at a concentration of microalgae biomass of 50 g/L. The maximum carbohydrate, 13.89 g/L, was obtained when a 1% acid solution and a 50 g/L biomass feed were used. After hydrolysis, the samples were cooled to room temperature and centrifuged at 7000 rpm for 5 min. The concentration of isolated total carbohydrates was determined by the phenol-sulfuric acid method.

Carbohydrates were isolated with the destruction of the microalgae cell wall. Microalgae cells are similar in structure to plant cells, but they have specific features due to the specificity of the habitat and metabolism. Microalgae cells have unique cell membrane features, which are multilayer, complexly organized structures [5], and include the plasmalemma, periplast, cell membrane, etc. The membranes of some algae species can secrete and accumulate substances on the cell surface, forming an additional hard cover, the cuticle, which contains carbohydrates [5]. It was found that all 3 types of carbohydrates added to the nutrient medium (glucose, fructose, maltose) had a positive effect on yield of *C. vulgaris* lipids. At the same time, fructose and maltose influenced *D. salina* lipid yield. The addition of carbohydrates to the medium had no effect on the lipid yield of *A. platensis*.

Extraction of total lipids was performed based on the Folch method, additionally the extraction of lipids by the Soxhlet method was studied [30]. The results correlated well with each other. The lipid species composition (neutral lipids, triacylglycerides, fatty acids, polar lipids, unsaponifiable substances, chlorophyllides, other impurities) were determined. Fatty acid identified in the lipid fraction were myristic acid, palmitic acid, oleic acid, stearic acid, linoleic acid. *C. vulgaris* samples contained 0.8–1.1% myristic acid, 15.8–17.2% palmitic acid, 18.4–22.0% oleic acid, 37.8–42.7% stearic acid, 6.9–8.3% linolenic acid; *D. salina* samples—2.2% myristic acid, 11.6% palmitic acid, 14.7% oleic acid, 23.6% stearic acid, 4.2% linoleic acid; *A. platensis* samples—0.6% myristic acid, 19.0–20.3% palmitic acid, 16.0–17.6% oleic acid, 43.8–45.7% stearic acid, 7.9–8.8% linoleic acid.

Lipid extraction from *Dunaliella salina* samples was the most efficient (above 25%). Our findings are consistent with previous research [29]. According to authors, the lipid extraction by the Folch method from *Dunaliella salina* biomass demonstrated the highest yield: 23.78% compared with that obtained using Soxhlet method (18.73%) [29]. Ahmed et al. [31] studied the lipid extraction from *Dunaliella salina* cultivated in a controlled environment for 21 days. The total lipids were 22.28%. This study suggested that high biomass and lipid production by *Dunaliella salina* can be achieved at a NaCl concentration of 2M in the culture medium [30]. According to HPLC analysis neutral lipids made up the majority of all lipids. *Arthrospira platensis* had the highest content of neutral lipids (above 58%). Stearic acid identified as the most common in all microalgae, but the greatest amount (more than 45%) was found in *Arthrospira platensis*. A study of the fatty acid composition of 35 *Arthrospira* strains was conducted [31]. Depending on the strain, linoleic, linolenic, and palmitic acids contents were, respectively, 13.1–31.5%, 13.1–31.5% and 42.3–47.6% of total fatty acids [31]. The reference [31] corresponds to *Arthrospira platensis*, and [29] to *Dunaliella salina* [29].

To visualize fats and chlorophyll, microalgae were stained after addition of glucose, maltose, and fructose to the nutrient medium. Lipids turned green, while chlorophyll turned orange (Figure 5, Figure 6 and Figure 7). The addition of one of the carbohydrates (maltose, fructose, or glucose) increased the accumulation of fatty acids in microalgae, particularly in *Chlorella*. Supplementation of culture medium with carbohydrates had lower on *Dunaliella* probably due to the initially high content of neutral lipids in this microalgae. The highest lipid content was observed in dense cultures with the addition of any carbohydrate. A further increase in the fat content had an inhibitory effect on the growth of microalgae.

*Chlorella* requires water, carbon dioxide, and a small amount of minerals for photosynthesis and reproduction. Excessive accumulation of lipids affects the processes of oxidation and photosynthesis and adversely affects the efficiency, duration, and energy consumption of the process of reproduction and growth of microalgae [5]. The addition of carbohydrates to nutrient media (Table 7), on average, in samples of all algae, led to an increase in the amount of neutral lipids by 10.9%, triacylglyceride by 10.9%, fatty acids by 13.9%, polar lipids by 3.1%, unsaponifiables by 13.1%, chlorophyllides by 12.1%, other impurities by 8.9%. The addition of carbohydrates to nutrient media (Table 8) in samples of all algae led to an increase in the amount of myristic acids by 10.8%, palmitic acids by 10.4%, oleic acids by 10.0%, stearic acids by 10.1%, linoleic acids by 5.7%.

The first step in the process of obtaining microalgae proteins was their lysis. The effectiveness of cell wall breakdown employing chemical reagents, physical (ultrasound), and enzymes was studied. The efficacy of one-stage and two-stage extraction of proteins from microalgae with different concentrations of hydrochloric acid and caustic alkali, either together or in alternate sequences, was investigated. The enzymatic method turned out to be the least efficient and, moreover, expensive due to the need to use three enzyme preparations simultaneously to obtain the best protein yield. This approach allowed obtaining 3–15% of protein on a dry matter basis.

For the first time, protein materials from *Dunaliella salina* and *Spirulina platensis* (*Arthrospira platensis*) were extracted and purified using fast protein liquid chromatography in a study [32]. They were then subjected to enzymatic hydrolysis by intestinal enzymes. The results showed that the extracted protein from *S. plantesis* (ProS) underwent faster hydrolysis than proteins from *D. salina* (ProD) due to their lower molecular weight and probably their greater flexibility and open structure. In addition, the degree of hydrolysis by trypsin and chymotrypsin of ProS was higher and faster than that of ProD due to the greater number of hydrolytic sites in ProS for both enzymes. The results showed that chymotrypsin can act better and faster than trypsin on peptide bonds of proteins, although the total of proteins after enzymatic hydrolysis was no more than 15% [32], as in our case. It was found that the protein recovery (%) gradually increased with increasing acid concentration in the extraction solutions. Although protein increased with increasing acid concentration, using a lower acid concentration was consistent with lower environmental impact and reduced operating costs. In pre-treatment, the protein was extracted more efficiently by concentrated alkaline solutions than by any concentration of hydrochloric acid, which is likely related to the hydrophobic nature of algae proteins (Table 3). Protein determination using the Kjeldahl method correlates well with protein content determined using the alternative method. Ultrasonication resulted in more efficient extraction when alkaline solutions and an ultrasonic length of 68.4 µm were used (Table 4). It is possible that the acid used before the alkaline treatment dissolved the cell wall polysaccharides, which facilitated the release of intracellular proteins.

Ultrasound with amplitude levels of 22.8 µm and 68.4 µm was studied as an unconventional pretreatment method to increase protein. An ultrasound amplitude of 68.4 µm applied to the rehydrated microalgae in an alkaline solution allowed obtaining a protein above 57%.

More advanced method of spray freeze drying, which combines two types of drying: spray and freeze drying has been reported [33]. Algae preparations are complex and must be handled carefully to remain effective. The relatively short shelf life of liquid microalgae suspensions, as well as the costs of storage and transportation during the cold season, necessitate the removal of water by drying. Although all drying methods have a common goal, they are conceptually different and not always suitable for working with temperature sensitive products. In addition, although most drying methods have been developed for batch processes, some, such as spray freeze drying (SFD), could theoretically be adapted to serve the continuous production of microalgae preparations. SFD is a technique that combines spray drying (which involves spraying a liquid to create finer particles) and freeze drying (which is especially important for drying thermally-sensitive materials) to produce dry powders with controlled size and increased stability [34]. It was found that a smooth temperature transition from the temperature of the protein eutectic (T = 31 °C) to room temperature 22 ± 0.2 °C, followed by a temperature transition to 0 °C is required to preserve the initial composition of the extracted protein complex. Furthermore, the time of cooling to 0 °C should not be shorter than 12 h. In order to avoid losses in the composition of the protein complex, we reduced the intensity of freeze drying (Table 6). In the first step of drying, the vacuum in the chamber was lowered to 30-35 Pa until the temperature reached the eutectic threshold. The vacuum was gradually increased after that (Table 6).

Microalgae are currently being seriously considered as a raw material for the production of biofuels. Since microalgae in the oceans account for more than 45 percent of global CO_2_ fixation [26], they are excellent candidates for the production of carbon-neutral biofuels. Active research is being conducted to increase the calorific value of cultivated microalgae. Due to an increase in the overall amount of neutral lipids up to 63%, certain species of *Chlorella* now have a calorific value of 29 kJ/g [29]. Finding conditions that increase the content of neutral lipids in microalgae is thus a major objective not just for introducing microalgae to functional foods, but also for creating fuels of the future.

## 5. Conclusions

The following microalgae have been isolated from natural sources (soil, water, sand) as a result of research: *Chlorella vulgaris* Beijer (1), *Chlorella vulgaris* Beijer (2), *Chlorella vulgaris* Beijer (3), *Arthrospira platensis* Gomont, *Arthrospira platensis* (Nordst.) Geitl. and *Dunaliella salina* Teod. The influence of different extraction methods on the proteins was studied. It was found that an ultrasound amplitude of 68.4 µm applied to rehydrated microalgae in an alkaline solution allowed obtaining a protein of more than 57%. The mineral residues, carbohydrates, proteins, and lipids contents was determined. The highest carbohydrate yield, 27%, was obtained from the dry biomass of *Chlorella vulgaris*. The effect of supplementation of nutrient medium by carbohydrates on the accumulation of lipids by mixotrophic metabolism was established. The lipid fraction included neutral lipids, triacylglycerides, fatty acids, polar lipids, unsaponifiable substances, and chlorophyllides. The composition of fatty acids included myristic, palmitic, oleic, stearic and linoleic acids. Therefore, establishing the effect of nutrient medium carbohydrates on lipid accumulation, studying the composition of lipids and fatty acids in microalgae is a promising direction for conducting diverse scientific research in the field of physiology, biochemistry, biophysics, genetics, space biology, etc. The study of the composition of microalgae is used to increase the productivity of water bodies and soil fertility, obtain biologically active substances, various food and feed additives, as indicator organisms in studying the current state of soils and water bodies [35].

## Figures and Tables

**Table 1:** Microalgae primer extrapairs.

Microalgae	Sequence (5′-3′)	Annealing Temperature, °C	Marker Gene	Source
*A. platensis*	Forward: CGGACGGGTGAGTAACACGTGA Reverse: GACTACTGGGGTATCTAATCCCATT	60	16s	[22]
*C. vulgaris*	Forward: ATTGGAGGGCAAGTCTGGT Reverse: ACTAAGAACGGCCATGCAC	55	18s	[23]
*D. salina*	Forward: GTCAGAGGTGAAATTCTTGGATTTA Reverse: AGGGCAGGGACGTAATCAACG	55	18s	[24]

**Table 2:** Proteins in different extraction methods.

Extraction Method	Microalgae	Extraction Type	Proteins (%)	Proteins According to Kjeldahl (%)
Enzymatic hydrolysis	1	Polysucrose degradation	6.90 ± 0.28	7.03 ± 0.20
	2	Polysucrose degradation	6.64 ± 0.28	6.83 ± 0.20
	3	Polysucrose degradation	6.90 ± 0.28	7.05 ± 0.20
	4	Polysucrose degradation	11.57 ± 0.08	11.96 ± 0.25
	5	Polysucrose degradation	10.63 ± 0.08	10.89 ± 0.23
	6	Polysucrose degradation	8.90 ± 0.23	8.93 ± 0.20
Physical treatment	1	Aqueous purification and potter homogenization	7.30 ± 0.21	7.32 ± 0.20
	2	Osmotic stress	6.77 ± 0.22	6.99 ± 0.20
	3	Osmotic stress	6.03 ± 0.22	6.26 ± 0.20
	4	Deep freeze	6.92 ± 0.12	7.10 ± 0.20
	5	Deep freeze	5.79 ± 0.12	5.96 ± 0.10
	6	Aqueous purification and potter homogenization	8.90 ± 0.23	8.97 ± 0.20
Chemical extraction	1	Acid-base treatment	59.76 ± 1.2	61.81 ± 1.2
	2	Alkaline and water treatment	69.34 ± 1.4	69.37 ± 1.4
	3	Alkaline and water treatment	63.68 ± 1.3	64.02 ± 1.3
	4	Acid-base treatment	59.54 ± 1.2	60.24 ± 1.3
	5	Acid-base treatment	55.26 ± 1.2	56.38 ± 1.2
	6	Acid-base treatment	79.73 ± 1.5	80.36 ± 1.5

1—*Chlorella vulgaris* C-11; 2—*Chlorella vulgaris* C-38; 3—*Chlorella vulgaris* C-66; 4—*Arthrospira platensis* B-256; 5—*Arthrospira platensis* B-287; 6—*Dunaliella salina* D-294. %—percentage of extracted material relative to the weight of the original sample.

**Table 3:** Characterization of chemically extracted proteins, carbohydrates, lipids.

Microalgae	Reagent	Average Molecular Weight (kDa)	Proteins (%)	Carbohydrates (%)	Lipids (%)
*C. vulgaris* C-11	0.1 M HCl	12.81	7.98 ± 0.23	7.12 ± 0.21	6.57 ± 0.19
	0.2 M HCl	13.04	7.75 ± 0.25	7.50 ± 0.23	6.81 ± 0.20
	0.3 M HCl	13.27	15.47 ± 0.11	8.37 ± 0.25	7.83 ± 0.23
	0.4 M HCl	12.62	16.90 ± 0.34	8.81 ± 0.26	7.56 ± 0.23
	0.1 M NaOH	12.87	51.47 ± 1.28	9.54 ± 0.29	9.12 ± 0.28
	0.2 M NaOH	12.82	50.31 ± 0.96	9.72 ± 0.29	9.33 ± 0.28
	0.3 M NaOH	12.82	49.25 ± 1.38	10.41 ± 0.31	9.98 ± 0.30
	0.4 M NaOH	12.82	56.42 ± 1.31	10.26 ± 0.30	10.7 ± 0.32
*C. vulgaris* C-38	0.1 M HCl	12.81	8.00 ± 0.47	7.37 ± 0.2	6.43 ± 0.19
	0.2 M HCl	13.09	7.15 ± 0.15	7.80 ± 0.23	6.96 ± 0.21
	0.3 M HCl	13.29	15.77 ± 0.21	8.62 ± 0.26	7.69 ± 0.23
	0.4 M HCl	12.66	16.80 ± 0.18	8.97 ± 0.27	7.50 ± 0.23
	0.1 M NaOH	12.86	51.38 ± 1.38	10.21 ± 0.31	9.37 ± 0.28
	0.2 M NaOH	12.84	50.73 ± 1.04	10.75 ± 0.32	9.65 ± 0.29
	0.3 M NaOH	12.85	49.47 ± 1.24	12.43 ± 0.37	9.83 ± 0.29
	0.4 M NaOH	12.88	56.62 ± 1.81	13.02 ± 0.39	10.92 ± 0.33
*C. vulgaris* C-66	0.1 M HCl	12.87	7.28 ± 0.67	7.23 ± 0.28	6.18 ± 0.20
	0.2 M HCl	13.03	7.34 ± 0.65	7.88 ± 0.28	7.18 ± 0.21
	0.3 M HCl	13.25	15.64 ± 0.65	8.73 ± 0.27	7.75 ± 0.23
	0.4 M HCl	12.67	16.24 ± 0.36	9.26 ± 0.30	7.91 ± 0.24
	0.1 M NaOH	12.87	51.46 ± 1.75	10.97 ± 0.31	9.88 ± 0.29
	0.2 M NaOH	12.86	51.46 ± 0.75	11.61 ± 0.42	9.97 ± 0.30
	0.3 M NaOH	12.84	49.41 ± 1.75	12.83 ± 0.50	10.16 ± 0.30
	0.4 M NaOH	2.83	56.64 ± 1.23	13.13 ± 0.51	10.93 ± 0.33
*A. platensis* B-256	0.1 M HCl	12.84	6.73 ± 0.12	9.46 ± 0.28	8.27 ± 0.25
	0.2 M HCl	13.43	6.74 ± 0.15	9.95 ± 0.29	8.89 ± 0.26
	0.3 M HCl	13.33	11.53 ± 0.12	10.93 ± 0.32	9.73 ± 0.29
	0.4 M HCl	12.43	17.42 ± 0.53	12.72 ± 0.37	9.91 ± 0.30
	0.1 M NaOH	12.51	34.46 ± 1.36	15.94 ± 0.51	10.74 ± 0.32
	0.2 M NaOH	12.51	30.41 ± 0.63	15.37 ± 0.50	11.47 ± 0.34
	0.3 M NaOH	12.49	39.32 ± 0.53	17.10 ± 0.52	12.19 ± 0.36
	0.4 M NaOH	12.84	66.41 ± 1.63	17.11 ± 0.52	12.96 ± 0.39
*A. platensis* B-287	0.1 M HCl	12.34	6.43 ± 0.18	9.62 ± 0.29	6.18 ± 0.18
	0.2 M HCl	13.65	6.85 ± 0.12	10.78 ± 0.32	6.73 ± 0.20
	0.3 M HCl	13.77	11.42 ± 0.12	12.37 ± 0.37	7.14 ± 0.21
	0.4 M HCl	12.87	17.63 ± 0.52	12.96 ± 0.39	7.67 ± 0.23
	0.1 M NaOH	12.23	34.63 ± 1.64	16.71 ± 0.50	9.51 ± 0.29
	0.2 M NaOH	12.42	31.1 ± 0.53	16.92 ± 0.51	9.82 ± 0.29
	0.3 M NaOH	12.43	39.78 ± 0.31	17.47 ± 0.52	10.42 ± 0.31
	0.4 M NaOH	12.53	67.4 ± 1.22	17.36 ± 0.52	10.99 ± 0.33
*D. salina* D-294	0.1 M HCl	12.65	8.20 ± 0.27	10.95 ± 0.33	4.57 ± 0.14
	0.2 M HCl	13.64	7.96 ± 0.28	11.93 ± 0.36	4.82 ± 0.14
	0.3 M HCl	13.43	14.91 ± 0.17	14.79 ± 0.44	4.98 ± 0.15
	0.4 M HCl	12.54	15.99 ± 0.14	16.34 ± 0.49	5.24 ± 0.17
	0.1 M NaOH	12.76	52.02 ± 0.27	18.38 ± 0.55	5.56 ± 0.18
	0.2 M NaOH	12.76	51.01 ± 0.21	18.96 ± 0.57	5.62 ± 0.18
	0.3 M NaOH	12.87	48.95 ± 0.38	20.22 ± 0.61	5.80 ± 0.19
	0.4 M NaOH	12.98	55.92 ± 1.21	20.71 ± 0.62	5.99.0 ± 0.20

%—percentage of extracted material relative to the weight of the original sample.

**Table 4:** Characterization of proteins, carbohydrates, and lipids obtained during joint extraction with acid and alkali.

Microalgae	Solvent	Average Molecular Weight (kDa)	Proteins (%)	Carbohydrates (%)	Lipids (%)
*C. vulgaris* C-11	0.4 M HCl → 0.4 M NaOH	13.27	59.34 ± 1.14	16.52 ± 0.50	7.71 ± 0.15
	0.4 M NaOH → 0.4 M HCl	13.81	52.32 ± 1.12	16.45 ± 0.49	8.93 ± 0.17
*C. vulgaris* C-38	0.4 M HCl → 0.4 M NaOH	13.17	59.76 ± 2.44	15.91 ± 0.48	8.11 ± 0.16
	0.4 M NaOH → 0.4 M HCl	13.92	51.07 ± 1.63	16.31 ± 0.49	9.38 ± 0.25
*C. vulgaris* C-66	0.4 M HCl → 0.4 M NaOH	13.75	59.21 ± 1.23	16.14 ± 0.49	8.44 ± 0.19
	0.4 M NaOH → 0.4 M HCl	13.56	51.43 ± 1.42	16.31 ± 0.49	9.71 ± 0.22
*A. platensis* B-256	0.4 M HCl → 0.4 M NaOH	13.44	61.76 ± 0.84	16.38 ± 0.49	10.10 ± 0.25
	0.4 M NaOH → 0.4 M HCl	13.83	58.07 ± 1.93	16.11 ± 0.49	11.08 ± 0.27
*A. platensis* B-287	0.4 M HCl → 0.4 M NaOH	13.37	61.64 ± 1.53	15.84 ± 0.47	9.46 ± 0.21
	0.4 M NaOH → 0.4 M HCl	13.84	58.64 ± 1.31	16.02 ± 0.48	9.63 ± 0.23
*D. salina* D-294	0.4 M HCl → 0.4 M NaOH	13.37	59.74 ± 1.53	15.95 ± 0.49	4.74 ± 0.13
	0.4 M NaOH → 0.4 M HCl	13.81	51.42 ± 1.42	17.19 ± 0.55	5.36 ± 0.14

%—percentage of extracted material relative to the weight of the original sample.

**Table 5:** The effect of ultrasound (US) on extraction of proteins, carbohydrates, and lipids.

Microalgae	Solvent and US Amplitude	Average Molecular Weight (kDa)	Proteins (%)	Carbohydrates (%)	Lipids (%)
*C. vulgaris* C-11	0.1 M HCl → US 22.8 µm	13.28	18.23 ± 1.32	16.13 ± 0.48	7.23 ± 0.16
	0.1 M HCl → US 68.4 µm	12.72	43.56 ± 1.65	16.05 ± 0.47	7.46 ± 0.16
	0.1 M NaOH → US 22.8 µm	12.89	26.48 ± 1.36	16.34 ± 0.49	7.77 ± 0.17
	0.1 M NaOH → US 68.4 µm	12.99	57.53 ± 0.31	16.76 ± 0.52	8.43 ± 0.19
*C. vulgaris* C-38	0.1 M HCl → US 22.8 µm	13.28	18.76 ± 1.34	15.83 ± 0.42	7.31 ± 0.16
	0.1 M HCl → US 68.4 µm	12.76	43.56 ± 1.54	15.86 ± 0.42	7.28 ± 0.16
	0.1 M NaOH → US 22.8 µm	12.89	26.66 ± 1.85	16.08 ± 0.47	7.67 ± 0.17
	0.1 M NaOH → US 68.4 µm	12.96	57.76 ± 2.33	16.41 ± 0.49	8.29 ± 0.19
*C. vulgaris* C-66	0.1 M HCl → US 22.8 µm	13.22	18.65 ± 1.65	15.57 ± 0.42	8.82 ± 0.21
	0.1 M HCl → US 68.4 µm	12.74	43.33 ± 2.53	16.28 ± 0.49	9.31 ± 0.23
	0.1 M NaOH → US 22.8 µm	12.86	26.34 ± 1.22	16.52 ± 0.50	9.68 ± 0.24
	0.1 M NaOH → US 68.4 µm	12.99	57.25 ± 2.35	16.60 ± 0.51	9.80 ± 0.26
*A. platensis* B-256	0.1 M HCl → US 22.8 µm	13.38	14.14 ± 1.32	16.84 ± 0.52	9.23 ± 0.22
	0.1 M HCl → US 68.4 µm	12.74	42.13 ± 2.21	16.91 ± 0.50	9.40 ± 0.28
	0.1 M NaOH → US 22.8 µm	12.85	23.27 ± 1.55	16.84 ± 0.49	9.71 ± 0.30
	0.1 M NaOH → US 68.4 µm	12.94	54.23 ± 2.31	17.29 ± 0.52	9.83 ± 0.30
*A. platensis* B-287	0.1 M HCl → US 22.8 µm	13.38	14.94 ± 1.02	16.22 ± 0.47	9.09 ± 0.26
	0.1 M HCl → US 68.4 µm	12.74	42.13 ± 2.21	16.27 ± 0.47	9.55 ± 0.28
	0.1 M NaOH → US 22.8 µm	12.83	23.27 ± 1.74	16.94 ± 0.48	9.82 ± 0.30
	0.1 M NaOH → US 68.4 µm	12.97	54.23 ± 2.31	17.61 ± 0.52	9.93 ± 0.31
*D. salina* D-294	0.1 M HCl → US 22.8 µm	13.22	18.94 ± 1.02	15.18 ± 0.41	4.56 ± 0.14
	0.1 M HCl → US 68.4 µm	12.76	43.13 ± 2.21	15.36 ± 0.42	5.42 ± 0.15
	0.1 M NaOH → US 22.8 µm	12.86	26.27 ± 1.74	15.49 ± 0.43	5.77 ± 0.15
	0.1 M NaOH → US 68.4 µm	12.97	57.23 ± 2.31	16.17 ± 0.60	5.95 ± 0.17

%—percentage of extracted material relative to the weight of the original sample.

**Table 6:** Content of proteins, carbohydrates, and lipids (%) in 100 g of dried microalgae without (A) and with (B) the addition of carbohydrates.

Organic Substances, %		Microalgae					
		*C. vulgaris*			*D. salina*	*A. platensis*	
		C-11	C-38	C-66	D-294	B-256	B-287
Proteins ^a^	A	51.0 ± 1.5	52.1 ± 1.5	51.8 ± 1.5	57.0 ± 1.4	50.3 ± 1.3	50.9 ± 1.3
	B	31.1 ± 0.9	34.2 ± 0.9	32.2 ± 1.0	34.2 ± 1.0	24.3 ± 0.7	12.4 ± 0.4
Lipids ^b^	A	12.7 ± 0.4	13.3 ± 0.5	13.9 ± 0.6	6.0 ± 0.2	16.1 ± 0.5 *	13.2 ± 0.4
	B	16.1 ± 0.2	17.1 ± 0.5	19.1 ± 0.6	25.4 ± 0.8	17.7 ± 0.5 *	17.7 ± 0.5
Carbohydrates ^c^	A	17.3 ± 0.7	16.0 ± 0.5	15.6 ± 0.4	32.0 ± 1.0	23.8 ± 0.8	26.3 ± 0.9
	B	41.6 ± 1.2	38.4 ± 1.1	43.3 ± 1.3	14.1 ± 0.4	43.9 ± 1.3	56.1 ± 1.7
Crude ash	A	19.0 ± 0.6	18.6 ± 0.6	18.7 ± 0.3	9.5 ± 0.3	9.8 ± 0.3	9.6 ± 0.3
	B	11.2 ± 0.3	10.3 ± 0.3	5.4 ± 0.1	26.3 ± 0.7	14.1 ± 0.4	13.8 ± 0.4

^a^ A nitrogen to protein conversion factor of 4.36 is used; ^b^ Standard deviations of triplicates; ^c^ The carbohydrate content is calculated by subtracting the lipid, protein, and ash content from 100% dry weight. %—percentage of extracted material relative to the weight of the original sample. Values in rows followed by the symbol “*” do not differ significantly (*p* > 0.05) as assessed by post hoc test (Tukey test).

**Table 7:** The lipid species composition of microalgae of 100 g microalgae without (A) and with (B) the addition of carbohydrates.

Lipids, %		Microalgae					
		*C. vulgaris*			*D. salina*	*A. platensis*	
		C-11	C-38	C-66	D-294	B-256	B-287
Neutral lipids ^a^	A	36.7 ± 1.1	34.1 ± 1.0 *	35.2 ± 1.1 *	57.3 ± 1.7	52.1 ± 1.6	58.2 ± 1.8 *
	B	39.3 ± 1.2	35.3 ± 1.1 *	36.2 ± 1.1 *	59.5 ± 1.8	54.6 ± 1.7	59.6 ± 1.8 *
Triacylglycerides	A	14.2 ± 0.4	15.2 ± 0.2 *	13.8 ± 0.2	26.4 ± 0.6	26.9 ± 0.3	51.1 ± 1.2
	B	16.1 ± 0.5	16.4 ± 0.6 *	15.8 ± 0.3	28.7 ± 0.6	28.4 ± 0.7	53.1 ± 1.4
Fatty acids	A	22.5 ± 0.7 *	18.9 ± 0.7 *	21.4 ± 0.7	30.9 ± 0.7	25.2 ± 0.7	7.1 ± 0.2
	B	23.9 ± 0.7 *	19.9 ± 0.7 *	23.2 ± 0.8	32.5 ± 0.9	27.2 ± 0.7	13.2 ± 0.4
Polar lipids ^b^	A	0.8 ± 0.02 *	0.7 ± 0.02 *	0.7 ± 0.02 *	16.4 ± 0.40*	0.9 ± 0.03 *	0.10 ± 0.03 *
	B	1.0 ± 0.04 *	0.9 ± 0.03 *	0.9 ± 0.03 *	18.6 ± 0.40 *	1.0 ± 0.04 *	0.30 ± 0.04 *
Unsaponifiables	A	13.1 ± 0.3	13.4 ± 0.3	13.2 ± 0.3	18.9 ± 0.5 *	16.2 ± 0.5	15.8 ± 0.5 *
	B	15.8 ± 0.5	15.9 ± 0.5	15.9 ± 0.5	20.5 ± 0.7 *	18.4 ± 0.6	17.2 ± 0.5 *
Chlorophyllides ^c^	A	5.1 ± 0.2	5.1 ± 0.2	5.2 ± 0.2	4.9 ± 0.1	14.6 ± 0.3	14.3 ± 0.3
	B	7.1 ± 0.2	7.1 ± 0.2	7.3 ± 0.2	6.5 ± 0.2	16.6 ± 0.6	16.7 ± 0.6
Other impurities ^d^	A	55.7 ± 1.7 *	47.7 ± 1.4	49.3 ± 1.5 *	2.5 ± 0.1	16.2 ± 0.5	4.5 ± 0.1
	B	56.3 ± 1.7 *	49.3 ± 1.4	49.3 ± 1.5 *	4.7 ± 0.1	18.3 ± 0.6	6.9 ± 0.1

^a^ Standard deviations of triplicates for triacylglycerides and fatty acids, and two repetition differences for unsaponifiables and polar lipids; ^b^ The polar lipids in this table are glycolipids and phospholipids quantified by HPLC, with a mass fraction of chlorophyll phytol side chains included in the unsaponifiables; ^c^ Chlorophyllide (the non-phytol fragment of chlorophylls) ws calculated based on the assumption that all chlorophyll pigments had the same molecular structure as chlorophyll a. d; ^d^ “Other impurities” is the difference between the 100% of dry weight of the original sample and the % of protein, lipid, and carbohydrate content. Values in rows followed by the symbol “*” do not differ significantly (*p* > 0.05) as assessed by post hoc test (Tukey test).

**Table 8:** Fatty acid composition of the lipid fraction of 100 g microalgae without (A) and with (B) the addition of carbohydrates.

Fatty Acids, %		Microalgae					
		*C. vulgaris*			*D. salina*	*A. platensis*	
		C-11	C-38	C-66	D-294	B-256	B-287
Myristic	A	1.1 ± 0.1 ^a^*	0.8 ± 0.1	1.0 ± 0.1 *	2.2 ± 0.1 *	-	0.6 ± 0.1 *
	B	2.3 ± 0.1 ^a^*	1.0 ± 0.1	2.4 ± 0.1 *	4.3 ± 0.1 *	3.6 ± 0.1 *	2.9 ± 0.1 *
Palmitic	A	15.8 ± 0.5	17.2 ± 0.6	16.1 ± 0.5	11.6 ± 0.3	19.0 ± 0.7	20.3 ± 0.9
	B	16.9 ± 0.6	18.7 ± 0.7	17.8 ± 0.6	13.6 ± 0.4	21.1 ± 0.8	22.3 ± 1.1
Oleic	A	18.4 ± 0.7	21.3 ± 0.9	22.0 ± 0.9	14.7 ± 0.5	17.6 ± 0.6	16.0 ± 0.5
	B	20.0 ± 0.9	22.8 ± 0.9	24.1 ± 1.0	16.1 ± 0.5	18.9 ± 0.7	18.1 ± 0.7
Stearic	A	40.5 ± 1.2	42.7 ± 1.3	37.8 ± 1.1	23.6 ± 0.9	45.7 ± 1.2	43.8 ± 1.1
	B	42.1 ± 1.2	44.9 ± 1.7	39.2 ± 1.1	25.1 ± 1.0	47.3 ± 1.3	45.6 ± 1.3
Linoleic	A	6.9 ± 0.2	7.2 ± 0.1	8.3 ± 0.2	4.2 ± 0.1	8.8 ± 0.2	7.9 ± 0.2
	B	7.2 ± 0.2	8.2 ± 0.2	9.0 ± 0.2	6.0 ± 0.2	9.9 ± 0.2	8.7 ± 0.2

^a^ Standard deviations of triplicates. Values in rows followed by the symbol “*” do differ significantly (*p* < 0.05) as assessed by post hoc test (Tukey test).

## Data Availability

The data are included in the manuscript.
